# Bioactive Endophytes Warrant Intensified Exploration and Conservation

**DOI:** 10.1371/journal.pone.0003052

**Published:** 2008-08-25

**Authors:** Stephen A. Smith, David C. Tank, Lori-Ann Boulanger, Carol A. Bascom-Slack, Kaury Eisenman, David Kingery, Beatrice Babbs, Kathleen Fenn, Joshua S. Greene, Bradley D. Hann, Jocelyn Keehner, Elizabeth G. Kelley-Swift, Vivek Kembaiyan, Sun Jin Lee, Puyao Li, David Y. Light, Emily H. Lin, Cong Ma, Emily Moore, Michelle A. Schorn, Daniel Vekhter, Percy V. Nunez, Gary A. Strobel, Michael J. Donoghue, Scott A. Strobel

**Affiliations:** 1 Department of Ecology and Evolutionary Biology, Yale University, New Haven, Connecticut, United States of America; 2 Department of Molecular Biophysics and Biochemistry, Yale University, New Haven, Connecticut, United States of America; 3 Herbario Vargas, Universidad Nacional San Antonio de Abad de Cusco, Cusco, Peru; 4 Department of Plant Sciences, Montana State University, Bozeman, Montana; Cairo University, Egypt

## Abstract

**Background:**

A key argument in favor of conserving biodiversity is that as yet undiscovered biodiversity will yield products of great use to humans. However, the link between undiscovered biodiversity and useful products is largely conjectural. Here we provide direct evidence from bioassays of endophytes isolated from tropical plants and bioinformatic analyses that novel biology will indeed yield novel chemistry of potential value.

**Methodology/Principal Findings:**

We isolated and cultured 135 endophytic fungi and bacteria from plants collected in Peru. nrDNAs were compared to samples deposited in GenBank to ascertain the genetic novelty of cultured specimens. Ten endophytes were found to be as much as 15–30% different than any sequence in GenBank. Phylogenetic trees, using the most similar sequences in GenBank, were constructed for each endophyte to measure phylogenetic distance. Assays were also conducted on each cultured endophyte to record bioactivity, of which 65 were found to be bioactive.

**Conclusions/Significance:**

The novelty of our contribution is that we have combined bioinformatic analyses that document the diversity found in environmental samples with culturing and bioassays. These results highlight the hidden hyperdiversity of endophytic fungi and the urgent need to explore and conserve hidden microbial diversity. This study also showcases how undergraduate students can obtain data of great scientific significance.

## Introduction

One argument for conserving biodiversity is that undiscovered species might yield products of great importance to humans. However, the link between undiscovered biodiversity and potential usefulness is largely conjectural. Here we provide direct evidence from bioassays of endophytes isolated from plants of tropical Peru and Bolivia.

Endophytes are fungi or bacteria that grow in the living tissues of plants, apparently without inflicting negative effects [1]. Each of the nearly 300,000 species of land plant on earth is likely to host to one or more endophyte species. Despite this anticipated diversity, relatively few of these organisms have been characterized. Many endophytes make bioactive natural products that inhibit the growth of other organisms, and in some cases they acquire the ability to synthesize the same defensive natural products produced by the plant [2], [3]. Consequently, endophytes are a potential source of novel products for use in medicine, agriculture, and industry. The enormous diversity of rainforest plants provides a vast untapped reservoir of potentially valuable endophytic organisms.

## Results and Discussion

During March 2007, S. Strobel and 15 undergraduate students in his HHMI-supported Yale course collected 304 vascular plant specimens from the Heath River area along the border of Peru and Bolivia, representing ∼200 species from ∼60 primarily angiosperm families. Small sections of the twigs of these plants were surface sterilized, dissected, placed on water agar [4], and pure cultures of the emerging endophytic fungi and bacteria were isolated. DNA was isolated from these cultures, and nuclear ribosomal (nr) gene-regions sequenced – the Internal Transcribed Spacer (ITS) region for fungi and 16S for bacteria (see Supporting Table S1).

**Figure 1:**
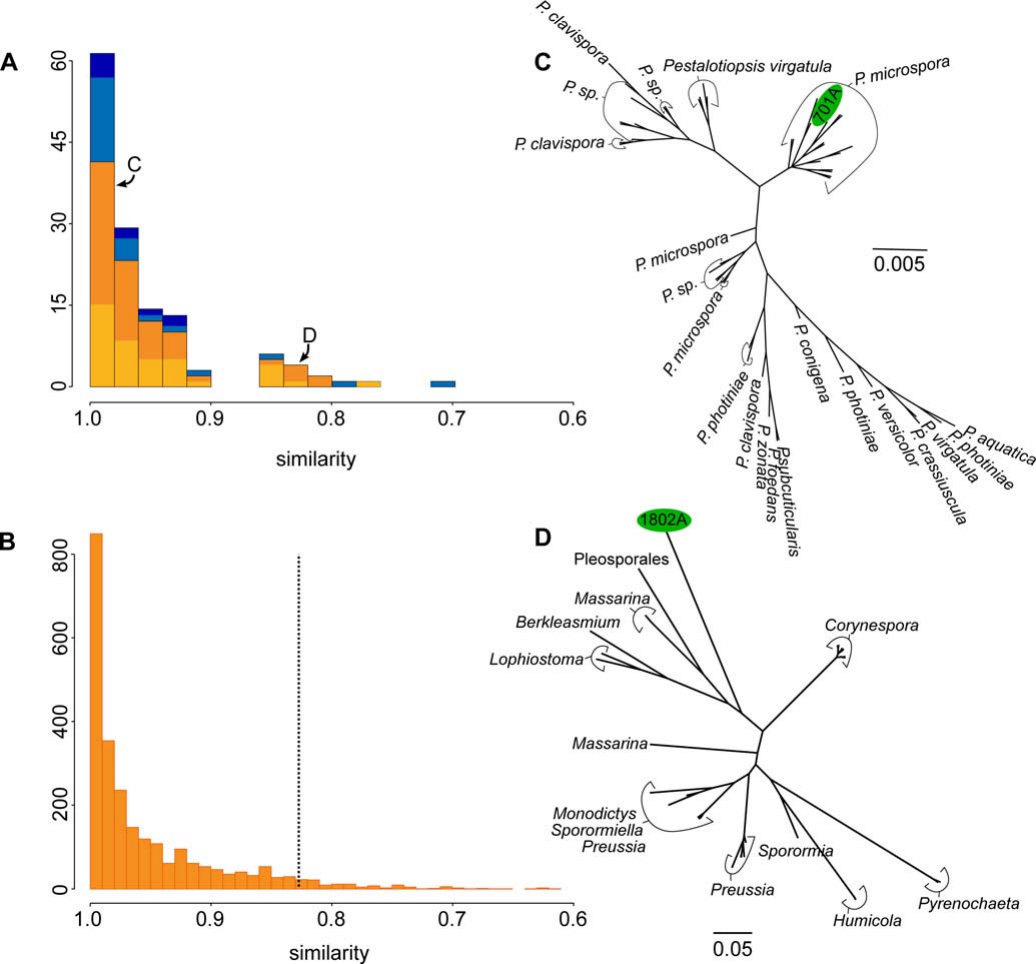
Endophytes diversity and GenBank. (A) The distribution of pairwise nrDNA ITS similarities between each cultured endophyte and its closest identified match in GenBank. The orange portions represent those identified as ascomycetes; blue portions represent other fungal and bacterial lineages. Lightened orange and blue portions indicate the proportion of endophytes identified as bioactive. “C” and “D” mark the positions of the sequences included in the trees in Figs. 1C and 1D. (B) The distribution of pairwise nrDNA ITS similarities for each ascomycete sequence in GenBank and its closest match in GenBank; those to the right of the dotted line fall in the top 5% of most divergent sequences. (C)&(D) Example of the phylogenetic position and evolutionary distances to the 40 most similar nrITS sequences in GenBank of two bioactive Peruvian ascomycete endophytes (cultures P701a and P1802a, in green).

BLAST searches of the nrDNAs obtained from 135 cultured endophytes were performed against GenBank (release 162) to assess their similarity to known sequences (Fig. 1A). Forty of the Peruvian endophytes differed by <1% from identified sequences in GenBank, 95 by <5%, and 120 by <10%; however, 10 differed between 15–30%. For ascomycete fungi (the group to which most of our endophytes belong; see below) we used Phyutility [5] to determine the similarity of each GenBank nrDNA sequence to the most similar sequence in GenBank (Fig. 1B). The dissimilarities of our endophytic sample of ascomycetes parallel the profile for the identified GenBank accessions. Importantly, several of our newly discovered ascomycetes are nearly as different from any GenBank accession as the most divergent known ascomycetes are from one another, and seven of them fall within the top five percent of the most divergent sequences known to date. These results support the suspected hidden hyperdiversity of endophytic fungi, and highlight our ignorance of fungal diversity in general [see 6].

We also included each endophyte nrDNA sequence in a phylogenetic analysis with the 40 most similar sequences found in GenBank (e.g., Fig. 1C, D). The major taxonomic group to which each endophyte sample belonged was determined by the assignment of the most similar identified sequence. Of the fungal endophytes, 101 appear to be ascomycetes, six are zygomycetes, and one is a basidiomycete. In addition, 14 bacterial endophytes were identified, of which eight are streptomycetes. The remaining 13 cultured endophytes clustered with unidentified sequences from environmental samples. Sequences quite similar to those present in GenBank fall squarely within recognized species and genera, and are separated by short branch lengths (e.g., Fig. 1C). Conversely, our highly dissimilar endophytes connect deeply and by long branches to known sequences (e.g., Fig. 1D).

In addition to bioinformatic analyses, the majority of the cultured endophytes were assayed for bioactivity. Each endophyte was placed in the center of a Petri dish and grown for ∼14 days. Agar plugs containing test organisms were placed adjacent to an endophyte and growth was assessed over 2–5 days. Test organisms included fungi, bacteria, and oomycetes, some of which cause plant and animal diseases (e.g., Candida albicans, Escherichia coli, Phytophthora sp.). Endophytes that inhibited the growth of any of the test organisms were scored as “bioactive.” Of the 135 endophytes, 88 were assayed, and 65 of these showed bioactivity (Fig. 1A).

As expected [e.g., 7], our survey of endophytes in tropical plants yielded major undiscovered diversity. Unlike other studies, however, our coupling of sequence analyses with bioassays demonstrates that many of the previously unknown microbes produce bioactive compounds. A number of the most dissimilar endophytes were among those that showed high levels of bioactivity. For example, Fig. 1D shows the phylogenetic placement of P1802a, an ascomycete isolated from Bauhinia guianensis (Fabaceae), which inhibited the fungus Fusarium and the oomycete plant pathogens Pythium and Phytophthora. This directly demonstrates the link between the discovery of novel biodiversity and novel chemistry of potentially great importance in medicine, agriculture, and industry.

Our results, combined with knowledge that biodiversity is rapidly being lost, highlight the immediate need for more deliberate exploration of biodiversity using similar approaches. The field and laboratory methods employed here are simple, inexpensive, and readily accessible to undergraduate students, yet have the potential to yield major surprises [8]. Extending such comparisons to other habitats in the same region and to similar habitats in different regions would shed light on the degree of host specificity and spatial patterns in phylogenetic diversity and bioactivity [cf. 9]. Finally, these findings underscore the need to conserve cryptic microbial diversity, not just for the sake of sustaining ecosystem services, but to maintain options for the beneficial use of biodiversity into the future.

## Materials and Methods

### Molecular methods

Total genomic DNA was extracted and purified from cultured endophytes using the QIAGEN Dneasy Plant Mini Kit following the protocols of the manufacturer (QIAGEN Inc., Valencia, California, USA). DNA for sequencing the regions of interest was generated via polymerase chain reaction (PCR) using the ITS1 and ITS4 primers [10] for the nrDNA Internal Transcribed Spacer (ITS) region from fungal endophytes and the 63F and 1387 primers [11] for the 16S nrDNA from bacterial endophytes. To ensure accuracy, both strands of the cleaned PCR products were sequenced using BigDye Cycle Sequencing Kit (Applied Biosystems, Foster City, California, USA) on an ABI 3730 DNA sequencer (Applied Biosystems, Foster City, California, USA) at the W.M. Keck DNA sequencing facility at Yale University.

### Sequence similarity

Each endophyte nrDNA was BLASTed against GenBank (word size = 28, match/mismatch scores 1/−2) with the closest taxonomically identified sequence returned (i.e., sequences returned from uncultured and/or unidentified environmental samples were removed from the analysis). A full local Smith-Waterman alignment was then conducted with the endophyte sequence and the GenBank sequence [12]. If the returned local alignment was shorter than the shortest sequence of the two, the alignment score was penalized accordingly; the amount unaligned in the shorter sequence was considered to be “mismatch.” For comparison to known fungal diversity, this same procedure was performed using all ascomycete nrITS sequences in GenBank. Each ascomycete was BLASTed against GenBank and the most similar sequence, excluding itself, was aligned and penalized as above (Fig. 1B).

### Evolutionary distance

To put the newly discovered endophyte diversity in an explicitly evolutionary context, each endophyte nrDNA was BLASTed against GenBank (word size = 28, match/mismatch scores 1/−2) and we used Phyutility [5] to retrieve the 40 closest taxonomically identified sequences, and from each of these sets of sequences we generated a phylogenetic tree (e.g., Fig. 1C, D). These sequences were aligned with Muscle 3.7 [13] and sites with more than 50% missing data were excluded from the phylogenetic analysis. Bayesian phylogenetic analyses were performed and summarized with MrBayes 3.1.2 [14]. Each Bayesian analysis was run for 106 generations and 105 generations were removed as burn-in. The branch lengths between the endophyte branch and the sister branch were then measured and reported using the majority rule consensus tree calculated from the posterior distribution of trees (Fig. S1). If the endophyte was sister to a clade of species, then the average distance from the endophyte to each tip in the clade was used.

### Bioassays

The organisms used to assay endophytic bioactivity were as follows: fungi: Botrytis sp., Candida albicans, Cerospora sp., Colletotrichum lagenarium, Fusarium solani, Geotrichum candidum, Rhizoctonia solani, Saccharomyces cervisiae, Sclerotinia sclerotiorum, Trichoderma viride, and Verticillium dahliae; bacteria: Bacillus subtilis, Escherichia coli, Klebsiella pneumoniae, and Staphylococcus epidermidis; oomycetes: Phytophthora cinnamomi and Pythium ultimum. Inhibitory activity of endophytes was scored as “none” when no inhibition was observed, “partial” if inhibition was between 0 and 100%. The percent inhibition was determined based on measurements of hyphal growth. For bacteria, estimates were made by eye, comparing streaks of bacteria on endophyte containing plates to controls lacking the endophyte.

## Supporting Information

Figure S1 Evolutionary Distance. Histogram showing the evolutionary distance of each endophyte from the most closely related sequence(s) as determined from phylogenetic analyses.
(0.47 MB TIF)

Table S1 Voucher and GenBank information. Plants collected and sampled for endophyte study are presented. GenBank accession numbers are also available.
(0.21 MB DOC)
