# Supplementary figures and images for: Bioactive Endophytes Warrant Intensified Exploration and Conservation
Source: PLoS One. 2008 Aug 25;3(8):e3052. doi: 10.1371/journal.pone.0003052 (PMC2518837; doi:10.1371/journal.pone.0003052)

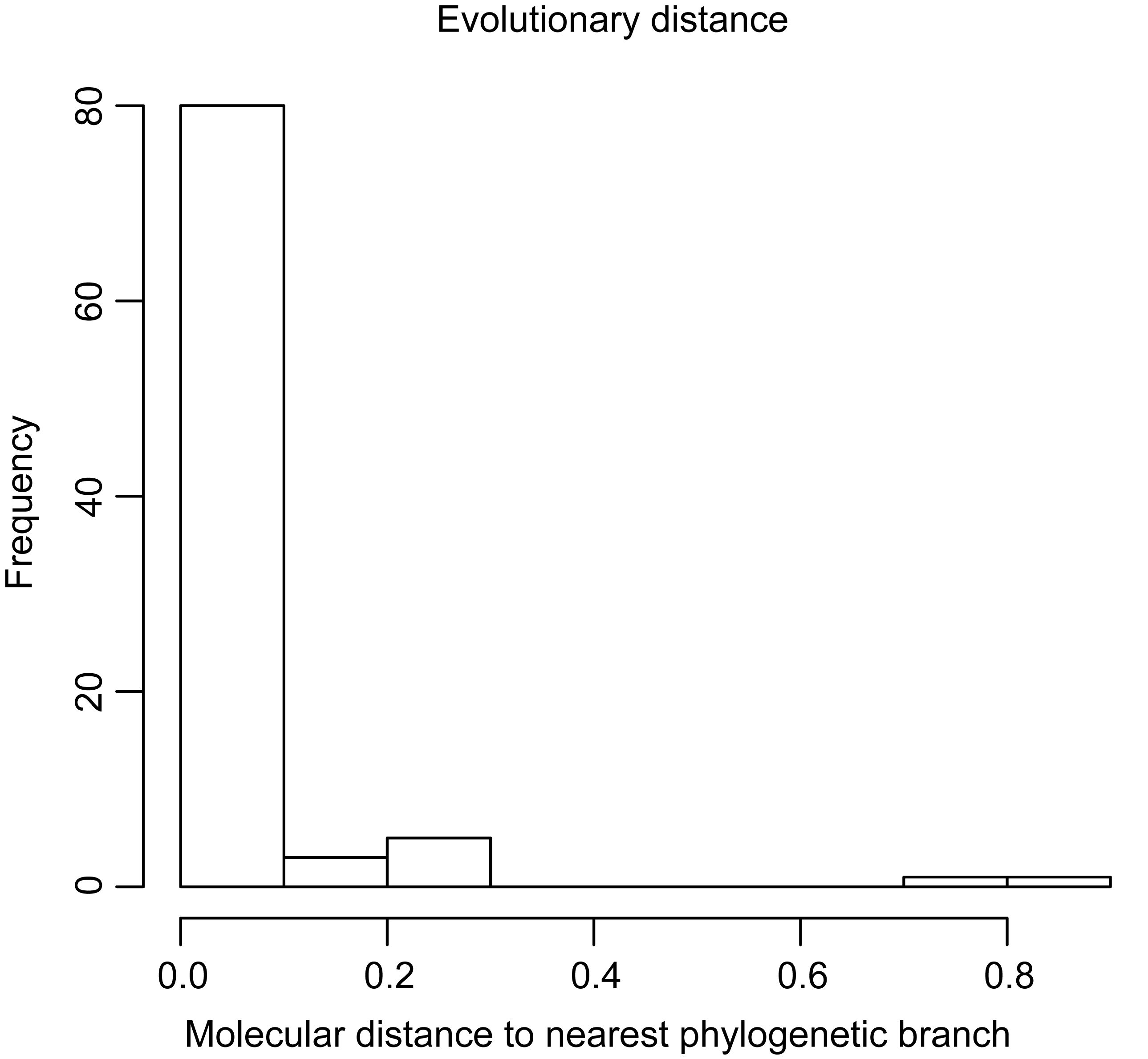

Supplement: Figure S1 — Evolutionary Distance. Histogram showing the evolutionary distance of each endophyte from the most closely related sequence(s) as determined from phylogenetic analyses. (0.47 MB TIF) [file pone.0003052.s001.tif]
