# Supplementary material for: Bioactive Endophytes Warrant Intensified Exploration and Conservation
Source: PLoS One. 2008 Aug 25;3(8):e3052. doi: 10.1371/journal.pone.0003052 (PMC2518837; doi:10.1371/journal.pone.0003052)
Supplement: Table S1 — Voucher and GenBank information. Plants collected and sampled for endophyte study are presented. GenBank accession numbers are also available. (0.21 MB DOC) [file pone.0003052.s002.doc]

Table S1. Voucher and GenBank information for the plant species and associated endophytes analyzed in this study. Plant and endophyte vouchers are deposited in the Yale University Herbarium (YU) in the Peabody Museum of Natural History.

| **Plant Family** | **Plant Species** | **Plant Voucher** | **Endophyte Voucher** | **GenBank #** |
| --- | --- | --- | --- | --- |
| Sapotaceae | *Pouteria bilocolaris* | A. Aguiar 101 | P101a2 | EU977266 |
| Monimiaceae | *Siparuna crassifolia* | B. Babbs 201 | P201b | EU977265 |
| Araceae | *Philodendron bipennifolium* | B. Babbs 210 | P210a | EU977296 |
| Dioscoreaceae | *Dioscorea trifida* | B. Babbs 213 | P213c | EU977209 |
| Droseraceae | *Drosera montana* | K. Eisenman 404 | P404aa | EU977277 |
|  |  |  | P404bb | EU977257 |
|  |  |  | P404c | EU977241 |
|  |  |  | P404e | EU977279 |
| Rubiaceae | *Calycophyllum acreanum* | K. Fenn 503 | P503b | EU977278 |
| Rubiaceae | *Capirona decorticans* | K. Fenn 506 | P506a | EU977222 |
| Rubiaceae | *Capirona decorticans* | K. Fenn 510 | P510a | EU977262 |
|  |  |  | P510c | EU977314 |
| Moraceae | *Brosimum alicastrum* | K. Fenn 512 | P512b | EU977226 |
|  |  |  | P512c | EU977255 |
| Lauraceae | *Ocotea longifolia* | K. Fenn 513 | P513a | EU977235 |
| Araceae | *Dracontium spruceanum* | K. Fenn 514 | P514a | EU977194 |
|  |  |  | P514b | EU977227 |
| Piperaceae | *Piper arboreum* | J. Greene 605 | P605f | EU977259 |
| Fabaceae | *Tachigalia polyphylla* | J. Greene 606 | P606a | EU977311 |
| Fabaceae | *Senna ruziana* | J. Greene 618 | P617a | EU977186 |
| Myristicaceae | *Iryanthera laevis* | B. Hann 701 | P701a | EU977206 |
| Araceae | *Monstera spruceana* | B. Hann 712 | P712d | EU977188 |
|  |  |  | P712e | EU977258 |
| Arecaceae | *Chamaedorea elegans* | B. Hann 713 | P713b | EU977219 |
| Rutaceae | *Ertela trifolia* | B. Hann 720 | P720a | EU977189 |
| Rubiaceae | *Palicoura sp.* | B. Hann 725 | P725a | EU977198 |
|  |  |  | P725f | EU977297 |
|  |  |  | P725g | EU977184 |
| Ericaceae | *Disterigma sp.* | B. Hann 753 | P753a | EU977240 |
|  |  |  | P753b | EU977203 |
| Apocynaceae | *Aspidosperma sp.* | J. Keehner 801 | P801a | EU977286 |
| Marantaceae | *Calathea poppigiama* | J. Keehner 802 | P802a | EU977238 |
|  |  |  | P802b | EU977221 |
|  |  |  | P802c | EU977221 |
| Fabaceae | *Tachigali polyphylla* | J. Keehner 806 | P806a | EU977190 |
| Araceae | *Anthurium clavigerum* | J. Keehner 807 | P807b | EU977202 |
| Rubiaceae | *Calycophyllum spruceanum* | J. Keehner 810 | P810a | EU977204 |
| Apocynaceae | *Himatanthus sucuuba* | J. Keehner 813 | P813c | EU977292 |
| Anacardiaceae | *Astronium graveolens* | J. Keehner 815 | P815a | EU977290 |
| Sterculiaceae | *Theobroma cacao* | J. Keehner 816 | P816a | EU977273 |
| Menispermaceae | *Chondrodendron sp.* | J. Keehner 818 | P818b | EU977298 |
|  |  |  | P818c | EU977310 |
| Rubiaceae | *Alibertia sp.* | E. Kelley-Swift 905 | P905a | EU977239 |
| Bignoniaceae | *Jacaranda copaia* | E. Kelley-Swift 908 | P908b | EU977242 |
| Rubiaceae | *Palicourea longifolia* | E. Kelley-Swift 911 | P911a | EU977303 |
|  |  |  | P911b | EU977307 |
| Rubiaceae | *Psychotria ernestii* | E. Kelley-Swift 912 | P912b | EU977236 |
| Malvaceae | *Hibiscus adscensionis* | E. Kelley-Swift 913 | P913a | EU977208 |
|  |  |  | P914a | EU977218 |
| Euphorbiaceae | *Jatropha curcas* | E. Kelley-Swift 916 | P916a | EU977224 |
|  |  |  | P919a | EU977271 |
| Monimiaceae | *Siparuna guanensis* | E. Kelley-Swift 919 | P919b | EU977275 |
| Malpighiaceae | *Banisteriopsis caapi* | V. Kembaiyan 1001 | P1001a | EU977212 |
| Apocynaceae | *Forsteronia affinis* | V. Kembaiyan 1004 | P1004a | EU977272 |
| Euphorbiaceae | *Manihot brachyloba* | V. Kembaiyan 1011 | P1011a | EU977263 |
| Urticaceae | *Urera baccifera* | V. Kembaiyan 1014 | P1014b | EU977251 |
| Bixaceae | *Bixa orellana* | V. Kembaiyan 1015 | P1015a | EU977299 |
|  |  |  | P1015b | EU977267 |
| Moraceae | *Ficus ypsilophlebia* | S. Lee 1201 | P1201a | EU977225 |
|  |  |  | P1201b | EU977192 |
|  |  |  | P1201c | EU977282 |
| Araceae | *Monstera spruceana* | S. Lee 1207B | P1207Bh | EU977223 |
| Olacaceae | *Minquartia guianensis* | S. Lee 1208B | P1208Ba | EU977287 |
| Araceae | *Geonoma piscicauda* | S. Lee 1209 | P1209b | EU977228 |
| Rhamnaceae | *Gouania rhamnsides* | S. Lee 1214 | P1214a | EU977245 |
| Dioscoreaceae | *Dioscorea acanthogene* | S. Lee 1223 | P1223a | EU977304 |
| Burseraceae | *Protium neglectum* | P. Li 1301 | P1301a | EU977294 |
| Araceae | *Monstera spruceana* | P. Li 1304 | P1304e | EU977230 |
| Rutaceae | *Ertela trifolia* | P. Li 1307 | P1307c | EU977291 |
|  |  |  | P1307d | EU977283 |
|  |  |  | P1307e | EU977185 |
| Pteridophyta | *Thelypteris lugubriformis* | P. Li 1308 | P1308c | EU977214 |
| Heliconiaceae | *Heliconia psittacorum* | P. Li 1309 | P1309b | EU977261 |
|  |  |  | P1309d | EU977211 |
|  |  |  | P1309f | EU977215 |
| Araliaceae | *Schefflera morotoni* | P. Li 1310 | P1310a | EU977193 |
| Fabaceae | *Senna herzogi* | P. Li 1311 | P1311d | EU977210 |
| Rubiaceae | *Palicourea triphylla* | P. Li 1312 | P1312a | EU977200 |
|  |  |  | P1312d | EU977268 |
| Rubiaceae | *Psychotria trivialis* | P. Li 1313 | P1313a | EU977243 |
|  |  |  | P1313f | EU977217 |
| Piperaceae | *Piper ostii* | P. Li 1315 | P1315f | EU977250 |
| Rubiaceae | *Uncaria guianensis* | P. Li 1316 | P1316c | EU977231 |
|  |  |  | P1316e | EU977248 |
| Polemoniaceae | *Cantua buxifolia* | P. Li 1318 | P1318e | EU977293 |
| Myristicaceae | *Iryanthera laevis* | D. Light 1403 | P1403g | EU977269 |
|  |  |  | P1403t | EU977316 |
|  |  |  | P1403u | EU977313 |
| Euphorbiaceae | *Acalypha mapirensis* | D. Light 1404 | P1404a | EU977260 |
|  |  |  | P1404d | EU977256 |
| Myristicaceae | *Iryanthera laevis* | D. Light 1406 | P1406h | EU977199 |
|  |  |  | P1406o | EU977237 |
| Melastomataceae | *Macairea thyrsiflora* | D. Light 1410 | P1410b | EU977233 |
| Polygalaceae | *Bredemeyera lucida* | D. Light 1412 | P1412a | EU977300 |
| Urticaceae | *Pourouma cecropiaefolia* | E. Lin 1501 | P1501a | EU977191 |
| Urticaceae | *Pourouma cucura* | E. Lin 1504 | P1504a | EU977220 |
|  |  |  | P1504b | EU977252 |
| Myrtaceae | *Calycolpus roraimensis* | E. Lin 1509 | P1509a | EU977187 |
| Solanaceae | *Nicandra physaloides* | E. Lin 1512 | P1512a | EU977315 |
| Myristicaceae | *Virola flexuosa* | E. Lin 1514 | P1514a | EU977295 |
| Lauraceae | *Ocotea longifolia* | E. Lin 1516 | P1516a | EU977289 |
| Amaryllidaceae | *Eucharis cyaneosperma* | E. Lin 1519 | P1519c | EU977285 |
| Myristicaceae | *Virola calophylloidea* | C. Ma 1605 | P1605a | EU977195 |
|  |  |  | P1605d | EU977247 |
| Rubiaceae | *Calycophyllum spruceanum* | C. Ma 1606 | P1606a | EU977306 |
| Euphorbiaceae | *Croton lechleri* | C. Ma 1612 | P1612a | EU977288 |
| Apocynaceae | *Himatanthus sucuuba* | C. Ma 1614 | P1614b | EU977312 |
| Rubiaceae | Unknown | C. Ma 1620 | P1620a | EU977276 |
|  |  |  | P1620b | EU977246 |
|  |  |  | P1620f | EU977264 |
|  |  |  | P1620g | EU977249 |
| Rubiaceae | *Uncaria guianensis* | C. Ma 1623 | P1623b | EU977270 |
|  |  |  | P1623d | EU977309 |
| Euphorbiaceae | *Jatropha curcas* | C. Ma 1624 | P1624b | EU977201 |
|  |  |  | P1624c | EU977213 |
| Rubiaceae | *Uncaria guianensis* | C. Ma 1628 | P1628a | EU977205 |
|  |  |  | P1628b | EU977253 |
| Violaceae | *Leonia crassa* | E. Moore 1701 | P1701b | EU977280 |
|  |  |  | P1701d | EU977216 |
|  |  |  | P1701f | EU977244 |
| Moraceae | *Naucleopsis krukovii* | E. Moore 1702 | P1702a | EU977232 |
| Fabaceae | *Senna sp.* | E. Moore 1708 | P1708b | EU977207 |
| Campanulaceae | *Hippobroma longiflora* | E. Moore 1717 | P1717c | EU977254 |
| Rubiaceae | *Palicourea guianensis* | E. Moore 1719 | P1719a | EU977196 |
| Fabaceae | *Bauhinia guianensis* | M. Schorn 1802 | P1802a | EU977274 |
| Unknown | Unknown | M. Schorn 1803 | P1803a | EU977284 |
| Unknown | Unknown | M. Schorn 1804 | P1804a | EU977234 |
|  |  |  | P1804b | EU977302 |
| Passifloraceae | *Passiflora sp.* | M. Schorn 1808 | P1808a | EU977317 |
| Rubiaceae | *Uncaria guianensis* | M. Schorn 1813 | P1813a | EU977308 |
|  |  |  | P1813b | EU977197 |
|  |  |  | P1813c | EU977301 |
| Sapindaceae | *Talisia reticulata* | S. Strobel 1903 | P1903a | EU977305 |
| Asteraceae | *Vernonanthura membranacea* | S. Strobel 1907 | P1907b | EU977281 |
| Apocynaceae | *Geissorpermum reticulatum* | S. Strobel 1921 | P1921a | EU977229 |
